# Porphyrin Production and Regulation in Cutaneous Propionibacteria

**DOI:** 10.1128/mSphere.00793-19

**Published:** 2020-01-15

**Authors:** Emma Barnard, Tremylla Johnson, Tracy Ngo, Uma Arora, Gunilla Leuterio, Andrew McDowell, Huiying Li

**Affiliations:** a Department of Molecular and Medical Pharmacology, Crump Institute for Molecular Imaging, David Geffen School of Medicine, UCLA, Los Angeles, California, USA; b Northern Ireland Centre for Stratified Medicine, Ulster University, Londonderry, United Kingdom; c UCLA-DOE Institute for Genomics and Proteomics, UCLA, Los Angeles, California, USA; University of Kentucky

**Keywords:** *Propionibacterium acnes*, propionibacteria, porphyrin, acne, skin microbiome

## Abstract

Porphyrins are intermediate metabolites in the biosynthesis of vital molecules, including heme, cobalamin, and chlorophyll. Bacterial porphyrins are known to be proinflammatory, with high levels linked to inflammatory skin diseases. Propionibacterium species are dominant skin commensals and play essential roles in defending against pathogens and in triggering an inflammatory response. To better understand how the inflammatory potential of the skin microbiome may vary depending on its propionibacterial composition, we compared the production levels of porphyrins among Propionibacterium acnes, Propionibacterium granulosum, Propionibacterium avidum, and Propionibacterium humerusii strains. We found that porphyrin production varied among these species, with P. acnes type I strains producing significantly larger amounts of porphyrins than P. acnes type II and III strains and other *Propionibacterium* species. P. acnes strains that are highly associated with the common skin condition acne vulgaris responded to vitamin B_12_ supplementation with significantly higher porphyrin production. In contrast, vitamin B_12_ supplementation had no effect on the porphyrin production of health-associated P. acnes strains and other propionibacteria. We observed low-level porphyrin production in most *Propionibacterium* strains harboring the *deoR* repressor gene, with the exception of P. acnes strains belonging to type I clades IB-3 and IC. Our findings shed light on the proinflammatory potential of distinct phylogenetic lineages of P. acnes as well as other resident skin propionibacteria. We demonstrate that the overall species and strain composition is important in determining the metabolic output of the skin microbiome in health and disease.

**IMPORTANCE** Porphyrins are a group of metabolites essential to the biosynthesis of heme, cobalamin, and chlorophyll in living organisms. Bacterial porphyrins can be proinflammatory, with high levels linked to human inflammatory diseases, including the common skin condition acne vulgaris. Propionibacteria are among the most abundant skin bacteria. Variations in propionibacteria composition on the skin may lead to different porphyrin levels and inflammatory potentials. This study characterized porphyrin production in all lineages of Propionibacterium acnes, the most dominant skin *Propionibacterium*, and other resident skin propionibacteria, including *P. granulosum*, *P. avidum*, and *P. humerusii*. We revealed that P. acnes type I strains produced significantly more porphyrins than did type II and III strains and other *Propionibacterium* species. The findings from this study shed light on the proinflammatory potential of the skin microbiome and can be used to guide the development of effective acne treatments by modulating the skin microbiome and its metabolic activities.

## Abstract (precis)

Porphyrins are a group of metabolites essential to the biosynthesis of heme, cobalamin, and chlorophyll in living organisms. Bacterial porphyrins can be proinflammatory, with high levels linked to human inflammatory diseases, including the common skin condition acne vulgaris. Propionibacteria are among the most abundant skin bacteria. Variations in propionibacteria composition on the skin may lead to different porphyrin levels and inflammatory potentials. This study characterized porphyrin production in all lineages of Propionibacterium acnes, the most dominant skin *Propionibacterium*, and other resident skin propionibacteria, including *P. granulosum*, *P. avidum*, and *P. humerusii*. We revealed that P. acnes type I strains produced significantly more porphyrins than did type II and III strains and other *Propionibacterium* species. The findings from this study shed light on the proinflammatory potential of the skin microbiome and can be used to guide the development of effective acne treatments by modulating the skin microbiome and its metabolic activities.

## INTRODUCTION

Over the last decade, with the advancements in microbiome studies, great strides have been made in our understanding of the microbial composition at different human body sites. In many cases, however, the functions and metabolic activities of these microbial communities and their interactions with the host are still not well understood.

Porphyrins are a group of metabolites involved in the biosynthesis of molecules essential to life (1, 2). Their characteristic tetrapyrrole ring-like structure allows them to bind metal ions such as iron and magnesium, facilitating their primary role as intermediates in heme and chlorophyll biosynthesis pathways, respectively (3). A build-up of bacterial porphyrins is also believed to play roles in human disease. Porphyrin levels on the skin of patients suffering from the common skin disease acne vulgaris (acne) are found to be significantly higher prior to successful treatment with antimicrobial therapies (4). The dominant porphyrin species produced by Propionibacterium acnes, coproporphyrin III, was shown to induce Staphylococcus aureus aggregation and biofilm formation in the nostrils (5). Porphyrin intermediates in bacterial heme biosynthesis pathways have been shown to induce inflammatory responses in human cells (6). Furthermore, coincubation of human keratinocytes with the synthetic coproporphyrin III tetramethyl ester has been shown to stimulate an increase in interleukin 8 (IL-8) expression after a 3-h incubation (6), supporting a role for porphyrins in skin inflammation.

We previously investigated the production of porphyrins by the dominant skin species P. acnes. P. acnes is classified into three subspecies and further into multiple phylogenetic clades that are known to differ in their virulence potential and associations with diseases (7–10). Comparison of porphyrin production from strains belonging to types I and II revealed significantly higher production levels in acne-associated strains (type I clade IA-2) than in strains associated with skin health (type II). This suggests that porphyrin production may be an important virulence mechanism helping to drive the inflammatory response seen in acne (8). We also observed that porphyrin production in P. acnes is modulated by host vitamin B_12_ levels, which influence the transcriptional activities of the skin bacteria *in vivo* (9). *In vitro*, vitamin B_12_ supplementation downregulates bacterial vitamin B_12_ biosynthesis and enhances porphyrin production in acne-associated clade IA-2 strains but not in health-associated type II strains (8, 9). Furthermore, comparative genomic analysis uncovered a *deoR* repressor gene upstream of the porphyrin biosynthesis operon in health-associated type II strains that is absent from acne-associated clade IA-2 strains (8). While it remains to be determined by what mechanisms P. acnes utilizes porphyrins, their production seems to correlate with the disease association and virulence property of P. acnes strains in acne.

Porphyrin production in P. acnes has been described in a number of independent studies, with each study often investigating a single type strain or a small number of clinically isolated strains (5, 6, 8, 11, 12). It is known, however, that individuals harbor complex skin microbial communities containing multiple *Propionibacterium* species and often a mixed P. acnes strain population (7). In fact, P. acnes strains from lineages that are not significantly associated with either acne or health (such as clades IA-1 and IB-2) represent the most prevalent and abundant strains in both acne patients and healthy individuals (7). In a recent study, we revealed that the balance between bacterial species and P. acnes strains is important when considering a role for the microbiome in skin health and disease. Beyond P. acnes, we also found that Propionibacterium granulosum was at a significantly higher relative abundance and increased prevalence in healthy individuals than in acne patients (13). Currently, porphyrin production and regulation in P. acnes strains beyond disease-associated (clade IA-2) and health-associated (type II) lineages have not been defined, as well as porphyrin production and regulation in other cutaneous, and potentially health-conferring, *Propionibacterium* species.

To better understand the contributions of the key members of the skin microbiome to health and disease, in this study, we selected representative P. acnes strains from all major lineages (type I clades IA-1, IA-2, IB-1, IB-2, IB-3, and IC, type II, and type III) and quantitatively compared their porphyrin production levels. Several other major human-associated propionibacteria, including P. granulosum, Propionibacterium avidum, and Propionibacterium humerusii were also analyzed. Furthermore, to correlate our quantitative *in vitro* measurements with clinical applications, we used Wood’s lamp, which is commonly used by dermatologists during skin examinations, to rapidly visualize porphyrin production across different *Propionibacterium* species and strains.

## RESULTS

### Porphyrin production is significantly higher in P. acnes type I strains.

**FIG 1:**
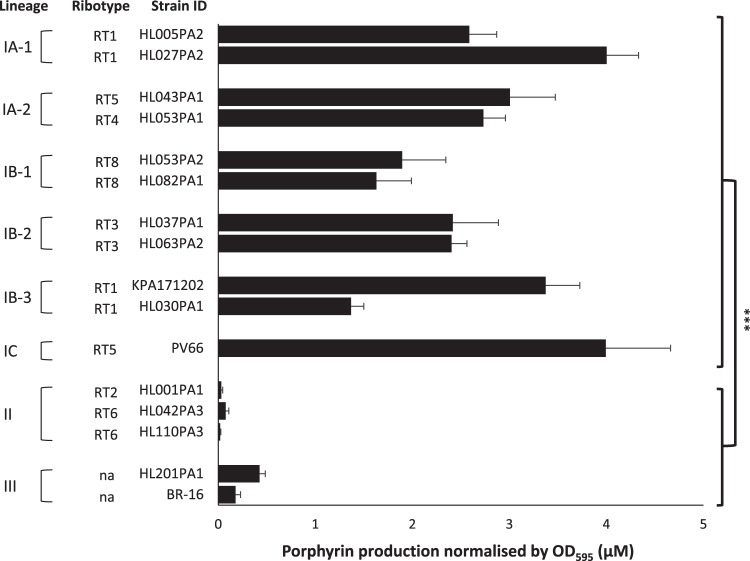
Porphyrin production across all known lineages of P. acnes. Porphyrin production was determined for 16 P. acnes strains representing all known lineages of P. acnes, as displayed on the left. Type I strains produced significantly more porphyrins than did type II and III strains. Porphyrin production is defined as the concentration of porphyrins produced (in micromolar) normalized by bacterial growth measured as the OD_595_. The average and the standard error of the mean (SEM) were calculated based on the data obtained from at least three independent experiments with at least four replicates each (***, *P < *0.001). ID, identifier; na, not assigned.

To compare porphyrin production across all major P. acnes lineages, we selected 16 strains representative of eight major phylogenetic groups, including type I clades IA-1 (*n* = 2), IA-2 (*n* = 2), IB-1 (*n* = 2), IB-2 (*n* = 2), IB-3 (*n* = 2), IC (*n* = 1), type II (*n* = 3), and type III (*n* = 2) (Table 1). We quantified the levels of secreted porphyrins produced by these strains (Fig. 1). While individual strains differed in their porphyrin production, type I strains consistently produced significantly higher levels of porphyrins, with an average of 2.67 μM (range, 1.4 to 4.0 μM), compared to a much lower average production of 0.04 μM observed in type II strains (range, 0.02 to 0.08 μM; *P = *5.2 × 10^−26^), and 0.37 μM observed in type III strains (range, 0.18 to 0.56 μM; *P = *9.8 × 10^−24^; Fig. 1).

**TABLE 1:** *Propionibacterium* strains used in this study^*a*^

Species	Clade	Ribotype	MLST_8_^*b*^	Strain name	Source/clinical association	*deoR*^*c*^
P. acnes	IA-1	RT1	ST15	HL005PA2	Facial skin	No
		RT1	ST1	HL027PA2	Facial skin	No
	IA-2	RT5	ST3	HL043PA1	Facial skin/acne	No
		RT4	ST3	HL053PA1	Facial skin/acne	No
	IB-1	RT8	ST4	HL053PA2	Facial skin/acne	No
		RT8	ST13	HL082PA1	Facial skin/acne	No
	IB-2	RT3	ST2	HL037PA1	Facial skin	No
		RT3	ST23	HL063PA2	Facial skin	No
	IB-3	RT1	ST5	KPA171202	Culture contaminant	Yes
		RT1	ST5	HL030PA1	Facial skin	Yes
	IC	RT5	ST85	PV66	Unknown/acne	Yes
	II	RT2	ST30	HL001PA1	Facial skin/healthy skin	Yes
		RT6	ST7	HL042PA3	Facial skin/healthy skin	Yes
		RT6	ST7	HL110PA3	Facial skin/healthy skin	Yes
	III	NA	ST123	HL201PA1	Tooth/endodontitis	Yes
		NA	ST75	BR-16	Back skin/PMH	Yes
*P. granulosum*	NA	NA	NA	HL078PG1	Facial skin	Yes
		NA	NA	HL082PG1	Facial skin	Yes
*P. avidum*	NA	NA	NA	HL063PV1	Facial skin	Yes
		NA	NA	HL083PV1	Facial skin	Yes
		NA	NA	HL307PV1	Facial skin	Yes
*P. humerusii*	NA	NA	NA	HL044PA1	Facial skin	Yes

a NA, not assigned; MLST, multilocus sequence type; ST, sequence type.

b Data from the MLST_8_ database (https://pubmlst.org/cacnes/).

c Homolog with *deoR* sequence PPA0299 from KPA171202.

### Acne-associated strains respond to vitamin B_12_ with increased porphyrin production, while other strains do not.

**FIG 2:**
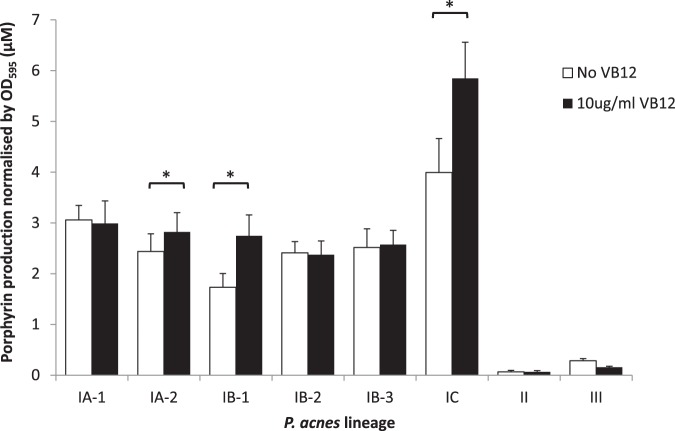
Acne-associated strains respond to vitamin B_12_ (VB12) supplementation and produce significantly more porphyrins. Strains from acne-associated P. acnes lineages (type I clades IA-2, IB-1, and IC) produced significantly more porphyrins when supplemented with vitamin B_12_ (10 μg/ml) than did the controls. Strains from other lineages (type I clades IA-1, IB-2, and IB-3, type II, and type III) did not respond to vitamin B_12_ supplementation in porphyrin production. Porphyrin production is defined as the concentration of porphyrins produced (in micromolar) normalized by bacterial growth measured as the OD_595_. Each bar represents the average porphyrin production by one or more strains in the same lineage. The average and the SEM were calculated based on the data obtained from at least three independent experiments with at least four replicates each (*, *P < *0.05).

We investigated and compared the effect of vitamin B_12_ on porphyrin production in all major P. acnes lineages, including acne- and health-associated strains. Previously, we revealed that acne-associated strains from clade IA-2 responded to vitamin B_12_ supplementation with increased porphyrin production (8). Acne association, defined by Fitz-Gibbon et al. (7) as an acne index, is assigned to P. acnes ribotypes (RT) based on the frequency of each ribotype identified in acne patients versus healthy individuals. A high acne index is representative of a ribotype identified with increased frequency in acne patients compared to healthy individuals. In addition to clade IA-2, strains from clades IB-1 and IC are also highly associated with acne (7, 14). We measured the porphyrin production from various P. acnes strains when supplemented with 10 μg/ml vitamin B_12_ in the culture (Fig. 2; see also Fig. S1 in the supplemental material). We found that strains from the acne-associated clades IA-2, IB-1, and IC responded positively to vitamin B_12_ supplementation with significantly increased porphyrin production compared to that in nonsupplemented controls (clade IA-2, *P = *0.038; clade IB-1, *P = *0.015; clade IC, *P = *0.050; Fig. 2). In contrast, strains from clades IA-1, IB-2, and IB-3, and in types II and III, which are not associated with acne, did not respond to vitamin B_12_ supplementation and did not produce more porphyrins when supplemented with vitamin B_12_ (Fig. 2). Thus, the differences in the overall abundances and ratios of multiple P. acnes strains in individuals may, in part, explain why only a subset of individuals are affected by vitamin B_12_-induced acne, as previously described (9, 15, 16).

10.1128/mSphere.00793-19.1 FIG S1 Strain-specific response to vitamin B_12_ supplementation. P. acnes strains from acne-associated lineages (clades IA-2, IB-1, and IC) produced significantly more porphyrins when supplemented with vitamin B_12_ (10 μg/ml; black bars) than did the controls (white bars). Strains from clades IA-1, IB-2, IB-3, II, and III did not respond to vitamin B_12_ supplementation with significantly increased porphyrin production. Each bar represents the average porphyrin level produced by each strain normalized by bacterial culture density (OD_595_). The average and standard error of the mean (SEM) were calculated based on the data obtained from at least three independent experiments with at least four replicates each (*, P < 0.05). Download FIG S1, PDF file, 0.1 MB. Copyright © 2020 Barnard et al. 2020 Barnard et al. This content is distributed under the terms of the Creative Commons Attribution 4.0 International license.

### Other *Propionibacterium* species produce significantly lower levels of porphyrins than P. acnes type I strains.

**FIG 3:**
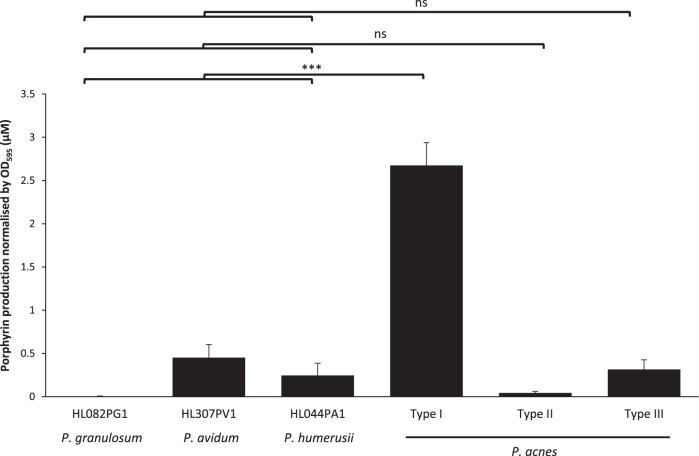
Other cutaneous propionibacteria produce significantly lower levels of porphyrins than P. acnes type I strains. Porphyrin production by three other *Propionibacterium* species, *P. granulosum* (HL082PG1), *P. avidum* (HL307PV1), and *P. humerusii* (HL044PA1), was determined and compared to the average levels of porphyrins produced by P. acnes strains from the types I, II, and III lineages. Other *Propionibacterium* species produced significantly lower levels of porphyrins than did P. acnes type I strains. Porphyrin production is defined as the concentration of porphyrins produced (in micromolar) normalized by bacterial growth measured as the OD_595_. The average and the SEM were calculated based on the data obtained from at least four independent experiments, with at least four replicates each shown (***, *P < *0.001; ns, not significant).

In addition to P. acnes, which is the dominant species found on sebaceous skin, individuals also harbor other cutaneous propionibacteria, including *P. granulosum*, *P. avidum*, and *P. humerusii* (7, 17). We determined the ability of these resident propionibacteria to produce porphyrins. We examined three *Propionibacterium* strains, *P. granulosum* HL082PG1, *P. avidum* HL307PV1, and *P. humerusii* HL044PA1 (Table 1), which were all isolated from human skin. We observed that all three strains produced low levels of porphyrins, similar to P. acnes type II and III strains, with an average porphyrin production of 0.30 μM (range, 0.14 to 0.52 μM, Fig. 3). This level is significantly lower than that of P. acnes type I strains (*P = *5.2 × 10^−23^).

To determine the effect of vitamin B_12_ on porphyrin production in other propionibacteria, we supplemented *P. granulosum*, *P. avidum*, and *P. humerusii* cultures with 10 μg/ml vitamin B_12_ and measured their porphyrin levels. There was no significant difference in porphyrin production compared to that in control cultures without vitamin B_12_ supplementation (Fig. S2). We further validated our findings using three additional strains, *P. granulosum* HL078PG1, *P. avidum* HL063PV1, and *P. avidum* HL083PV1. The results from these three additional *Propionibacterium* strains further support our conclusions of low porphyrin production by other *Propionibacterium* species regardless of the presence or absence of vitamin B_12_ supplementation (Fig. S2).

10.1128/mSphere.00793-19.2 FIG S2 Other cutaneous propionibacteria do not respond to vitamin B_12_. Porphyrin production of six additional non-P. acnes *Propionibacterium* strains was determined. Non-P. acnes *Propionibacterium* strains produced significantly lower levels of porphyrins than did P. acnes type I strains (see data in Fig. 3). The six strains did not increase porphyrin production when supplemented with vitamin B_12_ (10 μg/ml; black bars) compared to that with the controls (white bars). Two strains (HL078PG1 and HL083PV1) produced no detectable porphyrins in the presence or absence of vitamin B_12_ supplementation. Porphyrin production is defined as the concentration of porphyrins produced (in micromolar) normalized by bacterial growth measured as the OD_595_. The average and SEM were calculated based on the data obtained from at least one independent experiment with at least four replicates. Download FIG S2, PDF file, 0.1 MB. Copyright © 2020 Barnard et al. 2020 Barnard et al. This content is distributed under the terms of the Creative Commons Attribution 4.0 International license.

### Presence and expression of *deoR* in P. acnes strains and other propionibacteria.

In our previous study, we identified a *deoR* repressor element upstream of the porphyrin biosynthesis operon in P. acnes and suggested that it may play a role in controlling low levels of porphyrin production in *deoR*-harboring strains (8). To determine if the presence of *deoR* is correlated with low porphyrin production across different P. acnes strains and other *Propionibacterium* species, we searched for the *deoR* gene PPA0299 in all known genomes of P. acnes and other cutaneous propionibacteria. We found that the *deoR* element is present in P. acnes strains from type I clades IB-3 and IC, types II and III, as well as in strains representing *P. avidum*, *P. granulosum*, and *P. humerusii* (Table 1). Most *deoR*-harboring strains produce inherently low levels of porphyrins, including all tested type II and III P. acnes strains and other cutaneous propionibacteria. However, we observed moderate-to-high porphyrin production in P. acnes clade IB-3 and IC strains (range, 1.37 to 4.0 μM; Fig. 1). We tested for differences in *deoR* gene expression between P. acnes strains with high and low porphyrin production levels. We extracted RNA from log-phase cultures of three *deoR*-harboring strains, including two strains that produced high levels of porphyrins, KPA171202 (clade IB-3, 3.38 ± 0.4 μM) and PV66 (clade IC, 4.0 ± 0.67 μM), and one strain, HL201PA1, that produced naturally low levels of porphyrins (type III, 0.56 ± 0.11 μM). A *deoR*-negative strain, HL037PA1, was used as a negative control. We detected *deoR* expression in all three *deoR*-harboring P. acnes strains (Fig. S3). Since *deoR* was found to be expressed in both high- and low-level-porphyrin-producing strains, we sought to determine if there were differences in the *deoR* gene sequence that could explain variations in regulation and porphyrin production among the strains. We aligned *deoR* sequences from strains representing major P. acnes types as well as other cutaneous propionibacteria and constructed maximum likelihood phylogenetic trees (Fig. S4). While there were notable differences at the nucleotide level, we did not identify any single nucleotide polymorphisms (SNPs) that were uniquely shared by strains from clades IB-3 and IC and that could potentially explain the increased porphyrin production in these lineages. These results therefore suggest that additional factors likely play a role in the regulation of porphyrin production and the response to vitamin B_12_ supplementation.

10.1128/mSphere.00793-19.3 FIG S3 Strains from distinct P. acnes lineages carry and express *deoR*. P. acnes strains belonging to clades IB-3, IC, II, and III harbor *deoR*, a putative repressor gene located upstream of the porphyrin biosynthesis operon. Expression of *deoR* was detected via amplification of *deoR* fragment from the cDNA of KPA171202 (IB-3), PV66 (IC), and HL201PA1 (III). Strain HL037PA1, which does not carry *deoR*, is shown as a negative control. A housekeeping gene, *recA*, is used as a positive control for RNA extraction and PCR. Download FIG S3, PDF file, 0.1 MB. Copyright © 2020 Barnard et al. 2020 Barnard et al. This content is distributed under the terms of the Creative Commons Attribution 4.0 International license.

10.1128/mSphere.00793-19.4 FIG S4 Sequence similarity of *deoR* across *Propionibacterium* lineages. Maximum likelihood phylogenetic trees were constructed to show similarities of the *deoR* gene at the nucleotide (A) and amino acid (B) levels across *Propionibacterium* lineages. The P. acnes lineage or species name for each strain is listed, and IA, IB-3, IC, II, and III refer to P. acnes lineages. Download FIG S4, PDF file, 0.1 MB. Copyright © 2020 Barnard et al. 2020 Barnard et al. This content is distributed under the terms of the Creative Commons Attribution 4.0 International license.

### Confirmation of porphyrin production by propionibacteria using the Wood’s lamp.

**FIG 4:**
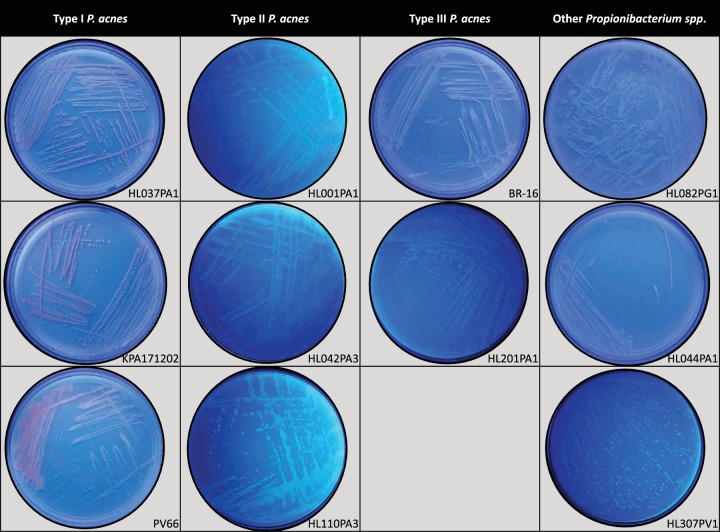
Wood’s lamp examination of porphyrin production of *Propionibacterium* strains cultured on solid medium. Eleven *Propionibacterium* strains were cultured on solid medium plates and illuminated with a Wood’s lamp. Detection of porphyrins was noted by orange-pink fluorescence from colonies. P. acnes type I strains that produce high levels of porphyrins appeared with a more intense orange-pink color than that with type II and III strains, as well as other *Propionibacterium* species, which produce low levels of porphyrins. Acne-associated clade IC strain PV66 produced the most pigment compared to other type I strains, consistent with the higher porphyrin levels determined by the extraction method (Fig. 1).

The Wood’s lamp is a dermatological tool used in the detection and management of many skin-related diseases, including microbial infections, acne, porphyria, and vitiligo (18). P. acnes fluoresces an orange-pink color when illuminated under a Wood’s lamp, owing to the production of porphyrins (19, 20). With our observations of different porphyrin production levels across propionibacteria species, we investigated if there were variations in the color appearance of different *Propionibacterium* species and strains when subjected to a Wood’s lamp examination, which potentially could be utilized in a clinic setting. We found that P. acnes type I strains producing moderate-to-high levels of porphyrins appeared orange-pink when plated on solid medium and illuminated with a Wood’s lamp. In particular, the acne-associated clade IC strain PV66 had the most intense orange-pink color observed, consistent with the highest level of porphyrins produced by this strain determined by the extraction method, compared to other type I strains tested (HL037PA1 and KPA171202; both clade IB-3). On the other hand, type II and III strains produced little, if any, orange-pink color; rather, a white color was observed for most of these strains, including the type III strains HL201PA1 and BR-16 (Fig. 4).

We also tested the appearance of *P. granulosum* and *P. humerusii* isolates under the Wood’s lamp. We found that, similar to the low-porphyrin-producing P. acnes strains, these strains also produced little to no orange-pink color (Fig. 4). The observation of different colors of *Propionibacterium* colonies when illuminated with a Wood’s lamp correlated well with the porphyrin levels quantitatively measured by the extraction method. These results further validate our conclusions that P. acnes type I strains contribute most of the porphyrins produced by skin bacteria.

## DISCUSSION

Porphyrins are proinflammatory metabolites that are believed to play a causal role in inflammatory skin conditions (4, 6, 21). Previously, we revealed that acne-associated P. acnes clade IA-2 strains produce significantly higher levels of porphyrins than do type II strains (8), which are associated with healthy skin (22). Since an individual can harbor a mixed P. acnes strain population and other *Propionibacterium* species on the skin, in this study, we sought to investigate the production of porphyrins across all major phylogenetic groups of P. acnes, as well as other resident cutaneous propionibacteria. Our results revealed that most type I strains produced a moderate-to-high level of porphyrins, in contrast to the little to no production in type II and III strains.

While P. acnes type I strains produce significantly more porphyrins than do type II and III strains (Fig. 1), only strains from acne-associated lineages (clades IA-2, IB-1, and IC) responded to vitamin B_12_ supplementation with significantly enhanced porphyrin production (Fig. 2). An increase in the production of proinflammatory porphyrins in strains from these lineages may help explain the association between these strains and acne. Acne patients harbor higher abundances of strains from clades IA-2, IB-1, and IC, compared to a low abundance or absence of these strains in healthy individuals (7, 13). In contrast, healthy individuals often harbor higher abundances of low-porphyrin-producing strains from the type II lineage (7, 13). Acne patients also tend to have higher serum vitamin B_12_ levels than do healthy individuals (23). Increased production of bacterial porphyrins upon high vitamin B_12_ levels may therefore lead to the phenomenon of vitamin B_12_-induced acne. Furthermore, individuals harboring complex skin microbial communities containing higher relative abundances of acne-associated strains, such as clades IA-2, IB-1, and IC, may be at an increased risk of acne development compared to individuals harboring lower abundances of these strains.

Compared to strains of types I and II, our understanding of the role of type III strains in human health and disease is much more limited. It has been suggested that P. acnes type III strains may play a role in the skin depigmentation disorder progressive macular hypomelanosis (PMH) (24–27) based on the higher prevalence and abundance of type III strains found in PMH lesions than other types, coupled with the visual observation of increased porphyrin levels detected by the Wood’s lamp method (20). Furthermore, when patients with PMH are treated with lymecycline and benzoyl peroxide, there is a reduction in the size of their lesions that appears to parallel a reduction in type III strains (26). In this study, using two P. acnes type III strains, one isolated from the lesional skin (back) of a PMH patient (BR-16) and the other from the oral cavity (HL201PA1), we found that both strains produced very low levels of porphyrins (Fig. 1). Low porphyrin production by these strains was further corroborated by the lack of significant pigment observed when these strains were cultured on solid medium and illuminated with a Wood’s lamp (Fig. 4). As skin often harbors multiple strains, with type I strains being the most common, this would likely indicate that the fluorescence observed within a PMH lesion is primarily driven by type I strains. Culture-based studies of punch biopsy specimens from lesional skin have, however, showed minimal evidence for mixed strain types (24, 27), but this could reflect the limitation and bias of *in vitro* culture. A recent metagenomic study, while corroborating the association of type III strains with PMH, demonstrated the presence of various amounts of type I and II strains in lesional samples in addition to type III strains. The analysis, however, was based on surface skin swab samples rather than biopsy specimens as in the previous study, and some potential issues around clear differentiation of lesional from nonlesional sites using this approach may have occurred (26). The differences among the studies demonstrate that further work is required to improve our understanding of the role of type III strains in PMH.

In addition to P. acnes, in this study, we investigated the porphyrin production of other cutaneous propionibacteria, including *P. granulosum*, *P. avidum*, and *P. humerusii.* We found that these species produce markedly low levels of porphyrins compared to P. acnes (Fig. 3). We also revealed that unlike acne-associated P. acnes strains, these cutaneous propionibacteria do not respond to vitamin B_12_ supplementation with respect to porphyrin production (Fig. S2). Recently, using both 16S rRNA and metagenomic shotgun sequencing analyses of samples collected from acne patients and healthy individuals, we revealed higher relative abundances of P. acnes type II strains, as well as *P. granulosum* in the skin microbiome of individuals with healthy skin (7, 13). Since individuals are often colonized by a community of mixed species and strain populations (7, 13), the ratios of different propionibacteria and P. acnes strains may ultimately determine the overall porphyrin production capability of an individual’s skin microbiome. A higher relative abundance of low-porphyrin-producing propionibacteria, such as P. acnes type II and *P. granulosum*, observed on healthy skin supports this theory, with an overall low production of proinflammatory porphyrins, thus maintaining a clinically healthy skin state.

The results from this study on the differential porphyrin-producing abilities of skin propionibacteria support and expand our previous findings. Using metagenomic analyses, we previously revealed that the balance of species, strains, and the overall functional content of the skin microbiome is important to skin health and disease. In this study, we quantitatively measured bacterial porphyrin production and highlighted the differences in the proinflammatory output from distinct *Propionibacterium* populations that are representative of the key players in the skin microbiome. The overall abundances of these distinct species and strains may ultimately define the combined metabolic output of the skin community, which is an important factor in the virulence potential of the resident skin microbiome in health and disease.

## MATERIALS AND METHODS

### Bacterial strains.

The *Propionibacterium* species and strains used in this study are listed in Table 1. Strains were isolated as previously described by Fitz-Gibbon et al. (7). HL005PA2 and HL027PA2 were selected to represent clade IA-1 strains, HL043PA1 and HL053PA1 were selected to represent clade IA-2 strains, HL053PA2 and HL082PA1 were selected to represent clade IB-1 strains, HL037PA1 and HL063PA2 were selected to represent clade IB-2 strains, KPA171202 and HL030PA1 were selected to represent clade IB-3 strains, PV66 was selected to represent clade IC strains, HL001PA1, HL042PA3, and HL110PA3 were selected to represent type II strains, and HL201PA1 and BR-16 were selected to represent type III strains. Other propionibacteria strains include *P. granulosum* HL078PG1 and HL082PG1, *P. avidum* HL063PV1, HL083PV1, and HL307PV1, and *P. humerusii* HL044PA1.

### Bacterial culture conditions.

Bacterial strains were cultured as described by Johnson et al. (8). Briefly, 5 ml of reinforced clostridial medium (RCM) was inoculated with 5 × 10^5^ P. acnes cells per ml of culture. Cultures were incubated anaerobically at 37°C in a light-protected box and grown to stationary phase (approximately 10 to 14 days). The growth of the cultures was monitored throughout the incubation period. For vitamin B_12_ supplementation, cultures were supplemented on day 0 with 10 μg/ml vitamin B_12_ (Sigma-Aldrich) and incubated as described above. Unless otherwise stated, three to eight independent experiments with at least four replicates per experiment were performed for each strain. Bacterial cultures with no vitamin B_12_ supplementation and medium controls were performed alongside the tests.

### Extraction and quantification of porphyrins.

Porphyrins were extracted according to the method described by Kang et al. (9). Briefly, 500 μl of stationary-phase bacterial cell culture was extracted in ethyl acetate and acetic acid (4:1 [vol/vol]) and solubilized in 1.5 M hydrochloric acid. The absorbance at 405 nm was measured from 200 μl of the soluble phase using a Genios M1000 spectrophotometer (Tecan U.S., Inc., Morrisville, NC). A standard curve was generated using coproporphyrin III standards (Frontier SCI) of known concentrations and used to convert absorbance to concentration. Bacterial culture density was measured as the optical density at 595 nm (OD_595_) for normalization of porphyrin levels between cultures.

### Statistical analysis.

The average level of porphyrins produced by each bacterial strain under each culture condition was calculated based on the data from at least three independent experiments, with at least four replicates for each trial, unless otherwise stated. Statistical significance between porphyrin levels produced by different strains or under different culture conditions (with vitamin B_12_ supplementation versus without supplementation) was analyzed using the Student's *t* test. All statistical analyses were performed using the R software (version 3.1.3).

### *deoR* repressor gene expression analysis.

The sequences of the *deoR* transcription repressor gene (PPA0299) found upstream of the porphyrin biosynthesis operon in a number of *Propionibacterium* strains (*deoR*-harboring strains) were aligned using the multiple-sequence alignment software MEGA7.0. To detect the expression of the *deoR* gene in P. acnes strains KPA171202, PV66, and HL201PA1, total RNA was extracted from log-phase cultures and purified using the RNeasy kit (Qiagen), according to the manufacturer’s instructions. DNA was removed using the Turbo DNA-free kit (Life Technologies). RNA quality and quantity were assessed by gel electrophoresis and NanoDrop spectrophotometer measurements. Single-stranded cDNA was synthesized using SuperScript III first-strand synthesis supermix (Life Technologies). PCR was performed using the C1000 thermal cycler (Bio-Rad), with the following primers: *deoR*-forward, 5′-CTGGCACGAGAAGGAACAA-3′; *deoR*-reverse, 5′-GAATCGAGCAGAACTAGGTCAC-3′; *recA*-forward, 5′-GTCGCGTTGGGTGTCGGAGG-3′; and *recA*-reverse, 5′-GATGCCGCCTTCAGCCTGGG-3′. The recA-forward and recA-reverse primers were used to analyze the expression of the housekeeping gene *recA* as a positive control. The PCR cycles are as follows: initial denaturation at 95°C for 5 min, followed by 35 cycles of 95°C for 10 s, 62°C for 30 s, and 72°C for 30 s, and then a final extension cycle at 72°C for 5 min. Amplified *deoR* and *recA* products were visualized on a 1.5% Tris-acetate-EDTA [TAE]–agarose gel. The *deoR*-negative P. acnes strain HL037PA1 (clade IB-2) was used as a negative control for *deoR* expression.

### Visualization of porphyrins using the Wood’s lamp.

Eleven *Propionibacterium* strains were cultured on solid A-medium plates, including P. acnes type I (HL037PA1, KPA171202, and PV66), type II (HL00PA1, HL042PA3, and HL110PA3), and type III (HL201PA1 and BR-16) strains, as well as *P. granulosum* (HL082PG1), *P. avidum* (HL307PV1), and *P. humerusii* (HL044PA1) strains. Plates were incubated for 5 to 7 days in a light-protected box. Wood’s lamp examination was conducted under dark-room conditions, with the lamp held approximately 20 cm from the medium plate.
