# Supplementary figures and images for: Porphyrin Production and Regulation in Cutaneous Propionibacteria
Source: mSphere. 2020 Jan 15;5(1):e00793-19. doi: 10.1128/mSphere.00793-19 (PMC6968654; doi:10.1128/mSphere.00793-19)

Figure S1

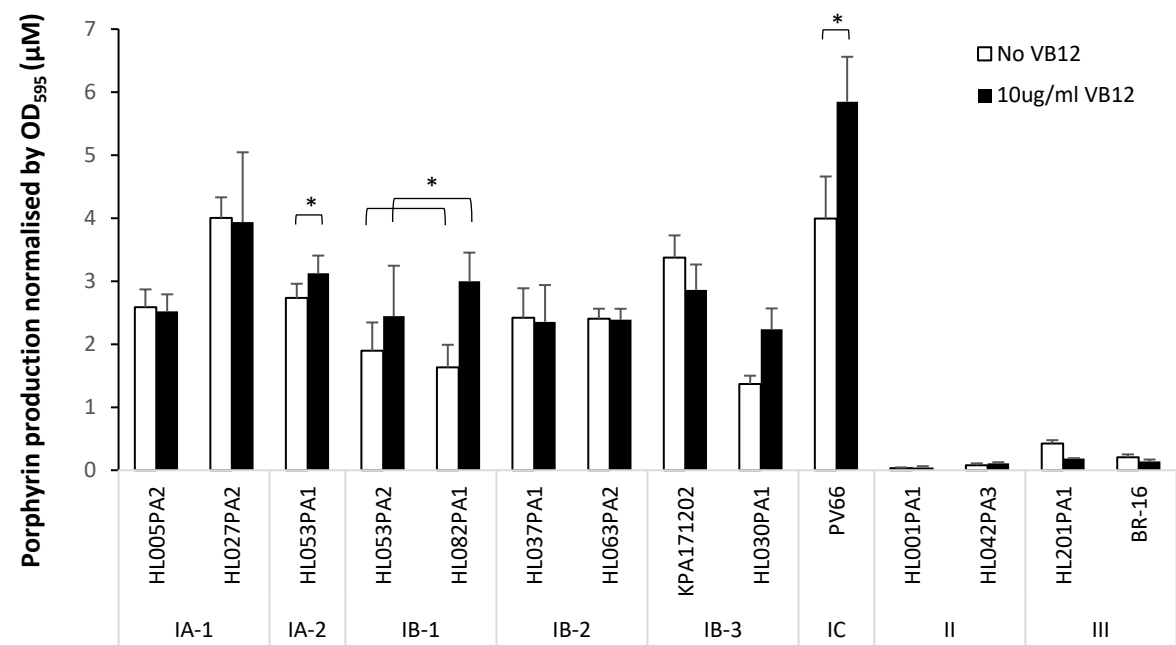

Supplement: FIG S1 [file mSphere.00793-19-sf001.pdf]

**Figure S2**

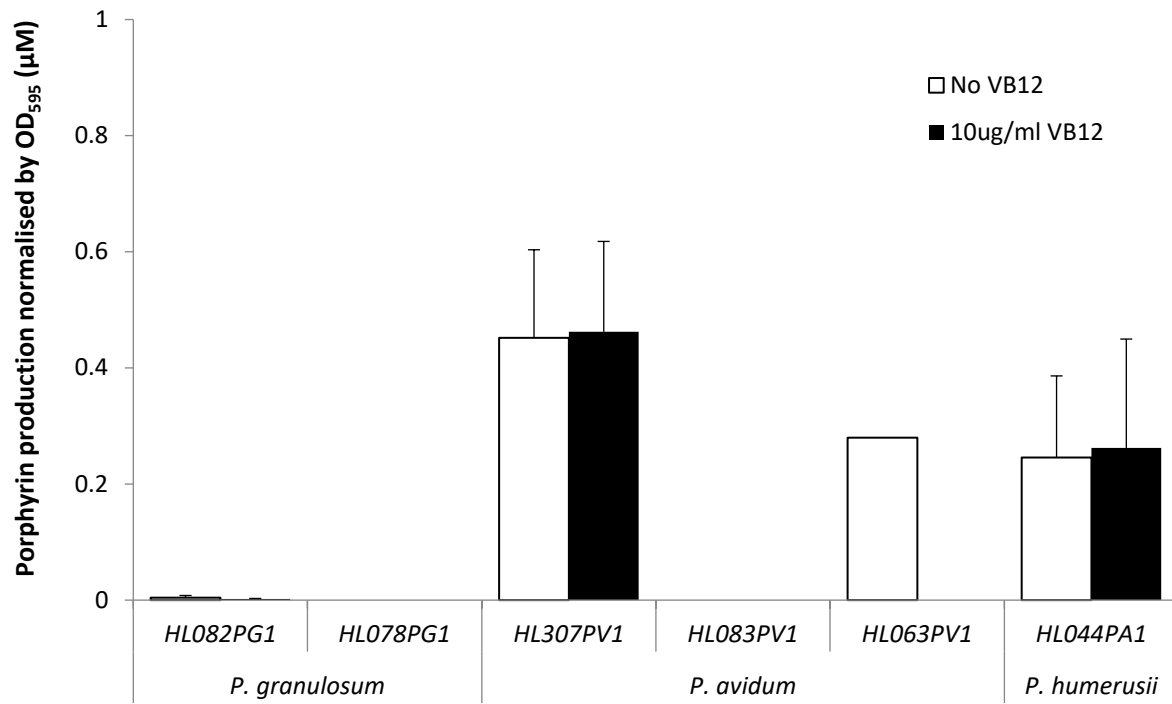

Supplement: FIG S2 [file mSphere.00793-19-sf002.pdf]

**Figure S3**

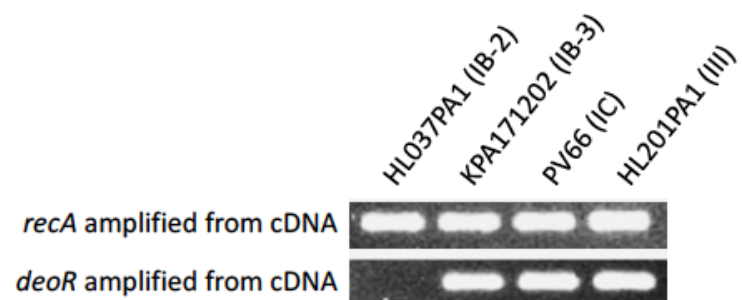

Supplement: FIG S3 [file mSphere.00793-19-sf003.pdf]

Figure S4

A

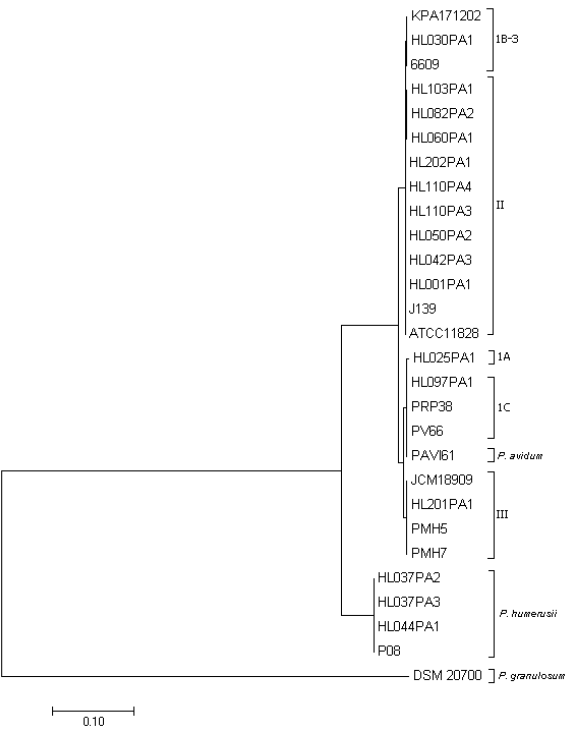

B

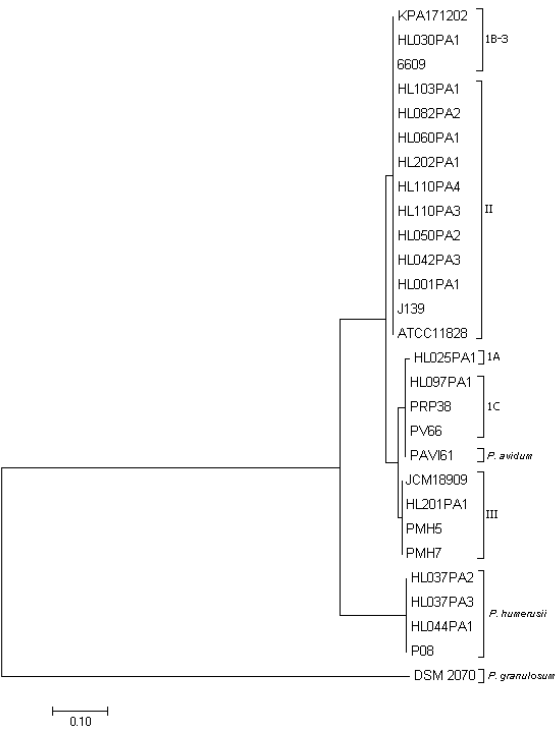

Supplement: FIG S4 [file mSphere.00793-19-sf004.pdf]
